# Exposure Detection Applications Acceptance: The Case of COVID-19

**DOI:** 10.3390/ijerph19127307

**Published:** 2022-06-14

**Authors:** Adi Alsyouf, Abdalwali Lutfi, Mohammad Al-Bsheish, Mu’taman Jarrar, Khalid Al-Mugheed, Mohammed Amin Almaiah, Fahad Nasser Alhazmi, Ra’ed Masa’deh, Rami J. Anshasi, Abdallah Ashour

**Affiliations:** 1 Department of Managing Health Services and Hospitals, Faculty of Business Rabigh, College of Business (COB), King Abdulaziz University, Jeddah 21991, Saudi Arabia; 2 Department of Accounting, College of Business Administration, King Faisal University, Al-Ahsa 31982, Saudi Arabia; 3 Health Management Department, Batterjee Medical College, Jeddah 21442, Saudi Arabia; mohammed.ghandour@bmc.edu.sa; 4 Medical Education Department, King Fahd Hospital of the University, Al-Khobar 34445, Saudi Arabia; mkjarrar@iau.edu.sa; 5 Vice Deanship for Quality and Development, College of Medicine, Imam Abdulrahman Bin Faisal University, Dammam 34212, Saudi Arabia; 6 Surgical Nursing Department, Faculty of Nursing, Near East University, Nicosia 99138, Cyprus; khalid.edu@yahoo.com; 7 Department of Computer Networks, College of Computer Sciences and Information Technology, King Faisal University, Al-Ahsa 31982, Saudi Arabia; malmaiah@kfu.edu.sa; 8 Department of Health Services and Hospital Administration, Faculty of Economics and Administration, King Abdulaziz University, Jeddah 21589, Saudi Arabia; fnalhazmi@kau.edu.sa; 9 Department of Management Information Systems, School of Business, University of Jordan, Amman 11942, Jordan; r.masadeh@ju.edu.jo; 10 Prosthodontics Department, Faculty of Dentistry, Jordan University of Science and Technology, Irbid 22110, Jordan; rjanshasi@just.edu.jo; 11 Department of Nursing, Faculty of Nursing, Philadelphia University, Amman 19392, Jordan; abodashour@yahoo.com

**Keywords:** exposure detection apps, tracing apps, mHealth, technology acceptance model, COVID-19

## Abstract

The pandemic’s context is rife with numerous dangerous threats and high fear levels, influencing human decision-making. Such characteristics are identified by investigating the acceptance of exposure detection apps from the technology acceptance model (TAM) perspective. This study purposed a model to investigate protection technology acceptance, specifically exposure detection apps in the context of COVID-19. Quantitative study approach and a cross-section design targeted 586 participants from Saudi Arabia. As the study model is complex, the study hypotheses were analysed using the structural equation modelling–partial least squares (SEM-PLS3) approach. The findings support the entire model hypothesis except the link between social media awareness and exposure detection apps’ intention. Mediation of COVID-19 anxiety and influence was confirmed as well. The current paper contributes to the technologies acceptance domain by developing a context-driven model comprising the major pandemic characteristics that lead to various patterns of technology acceptance. This study also fills the literature gap regarding mediating effects of social influence and COVID-19 anxiety in the relationship between trust in government and exposure detection apps implementation, and between COVID-19 anxiety and exposure detection apps implementation, respectively. The results may assist government agencies, health policymakers, and health organisations in the wide world and specifically Saudi Arabia, in their attempts to contain the COVID-19 pandemic spread.

## 1. Introduction

The novel coronavirus (COVID-19) disrupts several aspects of human life, including society, economy, and health [1,2,3]. The majority of public health institutions around the globe have attempted to search for control and reduction methods concerning the spread of COVID-19. The top effective strategies employed for surveillance and to contain the pandemic have been exposure detection apps—an app meant to assist public health institutions and authorities in addressing the transmission of COVID-19 from human to human by identifying individuals exposed to infected cases and informing them of the need to isolate themselves, carry out follow-ups, and undergo testing and treatment following the manifestation of symptoms [4,5]. Such apps can determine the number of new COVID-19 cases for every confirmed case and keep the numbers to a minimum. The advantage of such an app will only be realised if the public supports its usage. With the number of cases increasing, the app’s effectiveness will increase in determining potential cases and controlling and managing the spread of the virus [4].

Consequently, media and public health authorities have promoted the app among the citizens and residents for self-protection and contribution towards pandemic management worldwide. However, the use level is insufficient to effectively manage the proliferation of COVID-19 [6]. Thus, there is a need to understand how people decide to use an app through studies in order to suggest a list of practical interventions in the form of recommendations for maximised usage levels.

Commonly adopted technology adoption theories such as the unified theory of acceptance and use of technology (UTAUT) [7,8] and the technology acceptance model (TAM) have been used to portray the whole picture of how decisions are made regarding new technology usage (e.g., [4,9]). TAM is an influential model explaining technology usage behaviour [9,10]. Studies have validated the ability of the TAM model to shed light on the differences between the use of technology and behaviour towards technology and revealed it to be significant in predicting mHealth app usage to track individuals who have come in close contact with positive COVID-19 cases, essentially breaking the infection chain [4].

With the high rate of mortality and rate of infection [11], the concern is naturally high among people, along with the risk of being infected, motivating individuals to act and participate in self-protective behaviour, including the use of exposure detection apps [4,12,13].

Nevertheless, studies that addressed protection technology acceptance during the pandemic are still scarce; thus, providing an in-depth understanding of the situation would be beneficial. Accordingly, the study determines the answer to the question, “What factors explain the acceptance of exposure detection apps in the pandemic period?” This study resolves the question through an extended TAM, attempting to examine detection application usage when an individual comes in close contact with a COVID-19 case, after which an effective recommended response is provided. The study specifically added event-related fear, COVID-19 anxiety, trust in government, perceived privacy, social media awareness, and social influence for TAM extension.

During pandemics, risk and fear are generally the dominant emotions that arise. They are of top importance in understanding people’s engagement in self-protective behaviour. Unfortunately, only limited studies have been conducted to shed light on health protection technologies using TAM, except for Alsyouf et al. [4], who adopted TAM in addressing mHealth app usage to keep track of people who have had close contacts with positive COVID-19 cases. The study focused on the psychological determinants that stem from COVID-19 using a mobile health app based on the psychology field. The study also urged further studies to examine other related factors such as a lack of trust in the government and the way it deals with and confines the pandemic spread.

Every single day, people all over the world use an extensive range of social media mobile apps. According to Nabity-Grover et al. [14], there has been a considerable increase in social media platforms usage during the numerous lockdowns implemented because of the COVID-19 pandemic. In fact, social media apps have had a key role in the entertainment and reconnection of people, friends, and families during isolation, social distancing, and lockdown periods. Productivity apps have also indicated increased usage [15].

In this regard, a novel mobile app has been introduced as recommended by government authorities and health officials [16]. This type of app is an attempt of the government and the technology firms to confine the virus spread. In addition to the traditional manual contact tracing methods, the app helps in the rapid identification and tracking of individuals that have been in close contact with a positive case [17,18] and in promoting awareness concerning the virus through the provision of educational information about the infection and how it spread. However, most individuals remain concerned about using COVID-19 apps because of privacy issues, a lack of trust, and ethical concerns [19]. Moreover, several media platforms play a crucial role in shifting reality to raise more panic among the public, which makes them wary of using COVID-19 apps [20].

The public has used social media and productivity mobile apps at a high level during the pandemic [14,21]. Although social media has promoted the engagement and connection of people and increased information sharing, it has also promoted sensationalism. The spread of misinformation on the pandemic in the form of shocking and emotionally packed content is what people naturally gravitate to. Consequently, social media experts indicated that people have become increasingly anxious about what the future will bring, which has affected their adoption behaviours towards detection applications.

The extensive application of these technologies has led to privacy concerns and violations of civil liberties [22,23]. Most privacy and human rights preservation proponents have stated that the surveillance system development could cross boundaries of detection and warned that the gathered data could be utilised later to promote commercialisation [23,24].

Some have predicted that these information-gathering measures in periods devoid of pandemics could lead to problems that will hinder modern democracies, despite their appearance of usefulness at present and their necessity to confine the COVID-19 pandemic spread. This situation can be viewed in light of the “state of exception” [25], which is brought on by requirements stemming from the premise that particular laws must be established to preserve the current societal order. Such “exceptionalism” makes it possible to launch exceptions to present rights, including freedom of movement, freedom of assembly, and privacy, in order to preserve citizens’ security.

Such preservation of security was exemplified recently by the violation of individuals’ rights following the September 11 attacks in the United States in 2011. Although the U.S. government was lauded for its response to the threat, it was criticised for the “generalisation of the state of exception, through constant monitoring, surveillance and control of particular individual groups” [26].

During the pandemic, it seems that the state of exception and its potential outcomes have been discussed all over the globe. This begs the question of what drives people to adopt and accept risky surveillance technologies to protect against the pandemic? Do perceptions of the perceived threat and an individual’s coping skills in the pandemic affect their support for technological solutions? Does the importance of the relevant variables connected to the COVID-19 pandemic take precedence over the general attitudes towards liberty and authoritarianism?

Using COVID-19 tracing apps may be linked to several uncertainties, which can generally be categorised into health-related COVID-19 concerns and app-specific risks manifesting as performance risks and privacy risks that arise from the processing of sensitive data [27,28,29]. Additionally, social risks can arise among people due to their fear of social pressure/social exclusion from the use or non-use of the app [30].

In the field of information-seeking methods, social influence has a crucial role [31]. It is expected to mitigate the uncertainty people harbour regarding COVID-19 tracing apps. Basically, it is the level to which an individual views that people important to him are convinced that he should be using the system [32]. Individuals’ attitudes towards app usage may be influenced through information concerning their social environment preferences. Because COVID-19 tracing apps are primarily launched by governments in collaboration with government authorities and institutions, government trust has been investigated along with initial trust in COVID-19 tracing apps [29]. For the people to trust and use it, a specific level of transparency has to be reached in the COVID-19 tracing apps’ case [29]. Initial trust in COVID-19 tracing apps can be developed through the information quality they generate so long as past citizens-app interactions do not exist [33,34]. The fulfilment of specific information needs through the provision of clear and accurate information allows the initial trust of people to develop towards the technology, which, in this case, is the COVID-19 tracing app.

Hence, this study investigates the relevant variables of social media awareness, perceived privacy, social influence, and government trust as novel exogenous TAM predictors to shed light on the acceptance of exposure detection apps. The use of TAM in technology acceptance is aligned with the contextualisation approach that Alsyouf et al. [4] theorised. He contended that building IS theory calls for investigating the interconnection among the major context aspects within which the IS phenomenon occurs.

This study thereby contributes to the literature on technology acceptance in two ways. The first is examining protection technology acceptance in the context of the pandemic. The second is developing a context-driven model that examines the fear-risk relationship with perceived privacy, social media awareness, social influence, and intent to use exposure detection apps. The literature gap is filled concerning the mediating effects of social influence and COVID-19 anxiety on the trust in the government/exposure detection app use relationship and in between the event-related fear-exposure detection apps usage relationship. The proposed model contributes to the understanding of technology acceptance during the pandemic period and responds to the need and suggestions in literature to contextualise the IS research theories [4].

### 1.1. Literature Review

#### 1.1.1. Novel COVID-19 Coronavirus

The COVID-19 outbreak was first reported in Wuhan, Hubei province, China, in December 2019 [35,36]. It has claimed a significant number of lives and is spread extensively through human-to-human contact [37]. The chief characteristic of COVID-19, which is its fast spreadability, has led to the current global pandemic outbreak 2022 [38]. Based on medical research, the mortality rate caused by the pandemic has increased to 4% among infected individuals [39]. As a result, cases have risen to 363,834,233, and deaths have numbered 5,647,743 around the globe [40] at the time of this research.

Developing nations bore the brunt of the pandemic outbreak, which held true for the Middle East, particularly Saudi Arabia, where rapid COVID-19 spread has been experienced. According to the Saudi Ministry of Health (MOH), there have been 670,997 confirmed cases and more than 8929 deaths brought on by COVID-19 as of 27 January 2022 [40].

Owing to the COVID-19 rapid spread all over the globe, WHO and other public health authorities have adopted efforts and measures to confine it. Without proper vaccination, the initial responses depended on integrating public safety compliance to mitigate the epidemic reach. These included social distancing, wearing a facemask, and hygiene in one model (PSC triangle) [41]. In addition to scientists’ endeavours to develop an effective vaccine, policymakers in various countries have set up several measures such as contact tracing/exposure detection apps, through which potential transmission of COVID-19 among the population can be traced, assessed, isolated, and treated [4]. This enabled citizens and health authorities to contribute to controlling the pandemic’s spread. Apps developed for this purpose, such as the exposure detection app, have been directed towards keeping track of the number of COVID-19 cases and keeping it to a minimum. Such apps have several benefits but their full potential can only be achieved if most of the population adopts them for better virus spread management and control [4,42,43].

In a related simulation study, Hinch et al. [44] evidenced the effectiveness of exposure detection apps in mitigating infections if around 60% of the population accept and use them, and also, most public health agencies contended that such apps are not enough to ensure effective management of the virus if not adopted sufficiently by the majority [6]. Thus, it is crucial to study the factors influencing app usage decisions.

#### 1.1.2. Previous Research

Despite the protective strategy served by the use of exposure detection apps and their novelty, research circles have long been discussing the determinants of adopting protective behaviour through the use of theoretical assumptions. Such endeavours were carried out to shed light on the cognitive process used by an individual to decide whether or not to engage in protective behaviour.

A proportion of IS-dedicated literature has been directed towards protective behaviours, including mobile/online health and telemedicine adoption [45,46], compliance to information security policy [47], and others. In these studies, researchers have used two general theoretical methods to examine protective behaviours, with the first approach depending on general theories to shed light on and predict different general behaviour types, the top theories being the theory of reasoned action (TRA) and the theory of planned behaviour (TPB). Both theories have been used in investigating human behaviour such as protective behaviour (intention) and behavioural intention, stemming from attitude and social pressures [48].

More specifically, TPB contributes to the behaviour perception control effect on behavioural intention and actual behaviour [49]. Based on empirical findings, the key assumptions of TPB and TRA have been supported [50,51]. Adaptive behaviour calls for novel coping methods and technologies. Other contributors have relied on the well-known technology acceptance theories, including TAM and UTAUT, to explain the acceptance and use of technologies among individuals [4,52,53,54,55,56,57]. Notwithstanding the various terms used to describe drivers of new technology acceptance, the theories have been used to predict new technology adoption. They mainly use effort and performance expectancy constructs, facilitating conditions, and social pressures (e.g., [7,8,9,31,32]).

The TAM is an effective adoption model in management and information sciences, highlighting and explaining the drivers and technology acceptance and use [58]. TAM initially stemmed from its predecessor, a psychology-based theory, the theory of reasoned action (TRA). The latter model explains the relationships between the individual’s beliefs, attitudes, and intentions and their actual performance/non-performance of a certain behaviour [48]. According to the TRA, the attitude of the person can predict his actual behaviour, with attitude being the level of his positive/negative feelings regarding the concerned behaviour ([59] p. 984), subjective norms (significant people in his life and their expectations), and his intention (the level of intention towards performing the behaviour) ([59], p. 984). The extended TRA introduced by Davis et al. [60] was used to examine computer use behaviour as a particular case based on the assumption that TRA was introduced to explain every behaviour type.

The primary assumption of TAM states that technology use determinants are behavioural intention, which is affected by the technology’s perceived usefulness and ease of use [58], whereby an individual who thinks that technology is not easy to use and useful would likely refrain from using it, while another who finds it easy to use and useful would use it ([61] p. 2). In this study, TAM is considered the appropriate model for examining usability and usefulness effects on adopting exposure detection apps during the COVID-19 pandemic.

### 1.2. Theoretical Foundation

TAM was selected as the underpinning theory of this study—it is an influential and effective model in explaining technology usage behaviour [4,9]. According to TAM, the technology use behaviour (behavioural inclination towards technology acceptance) can be gauged using the individual’s technology usage attitude. There are two major predictors of attitude towards usage: perceived usefulness and perceived ease of use. The former represents the individual’s belief that technology use can promote task performance, whereas the latter represents the individual’s perception that it is easy to use the technology [59]. Added to this, there is an indirect effect of perceived ease of use on attitudes connected to perceived usefulness. TAM has been proven, time and again, to be effective in explaining the differences between technology use behaviour in various contexts, e.g., [62,63,64].

Regardless of the extensive examination of the models and their validation in the health information systems field among medical staff, their examination is limited when it comes to acceptance by consumers of health information applications [65,66,67]. Evidence shows that consumers’ acceptance of health informatics applications may differ from the acceptance of the same from professionals as the former may lack self-efficacy or use experience, which would mean that they are likely to face challenges when using an application [65,68,69]. Thus, finding ways to assist app acceptance among consumers is crucial.

Like other technology acceptance models, TAM has its drawbacks, among which is determining an individual’s attitude by other factors (e.g., social influence). An attitude of an individual towards IT use can be gauged using social influence [70,71]. TAM has been primarily used to examine internal motivations but not external ones, as it mainly focuses on the outcomes of IT usage. This means that the use process has not been entirely discussed, necessitating the extension of the model with external factors. As a result, it is highly recommended to extend the TAM model with external factors based on the conceptual model itself in order to add new variables to the TAM model. As such, to explain exposure detection apps adoption, this research adds psychological determinants associated with the COVID-19 pandemic (health anxiety sensitivity, event-related fear) to TAM as well as social media awareness, perceived privacy, social influence, and trust in government. In this regard, anxiety and fear are two emotional feelings that are significantly related to risks and can lead to emergencies. As a result, public health emergencies may arise. Both of these emotions imply pessimistic risk assessments. Therefore, it is imperative to study how fear and anxiety influence attitudes toward innovation, specifically with regard to an exposure app that will assist in monitoring and containing the COVID-19 pandemic.

On the other hand, social media use could have a significant impact upon health behavioural changes. This is due to mechanisms that increase fear in the public’s hearts by including pandemics in social media and encouraging them to adopt preventive behaviours. Thus, there is a need to investigate the relationship between social media alerts and exposure detection apps in order to limit COVID-19’s spread. Furthermore, the issue of privacy has repeatedly been brought up by users of COVID-19 apps. The situation is similar for the media and human rights organisations, which frequently oppose government initiatives to develop such apps for use by all members of society.

In general, trust reduces uncertainty and risk. Psychologists typically describe it as an intention to accept vulnerability based on positive expectations for another’s intentions or behaviour. However, trust may also be altered by actual app usage, becoming either stronger or weaker depending on user experiences. Further, individuals’ trust in their government also contributes to mitigating the uncertainty they face. The majority of COVID-19 tracing apps were developed by governments. Therefore, trust in the government may mitigate concern related to these apps. 

Social influence is an active information-seeking strategy. It refers to the degree to which an individual perceives that other individuals significant to him are convinced that he should adopt the proposed system. An analysis of individuals’ preferences concerning the social environment can reveal their attitudes toward the use of apps. To this end, this study integrates additional external variables for technology usage perceptions. Such integration has not been considered before, and attempts to reflect some of the aspects of the users that play a key role in successful technology usage.

**Figure 1:**
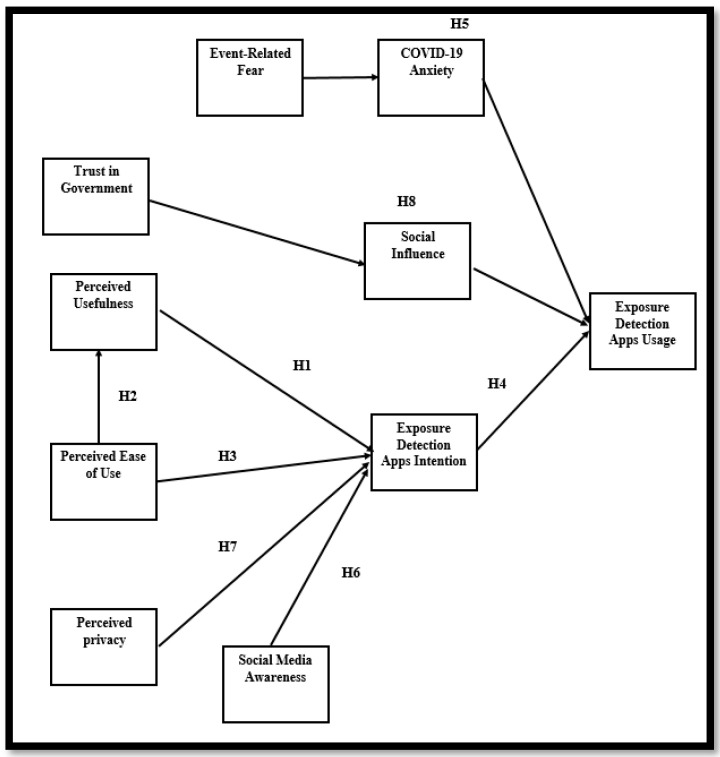
Research framework.

Figure 1 presents a graph representing the formulated hypotheses of the study.

#### Proposed Model and Hypotheses Formulation

##### Perceived Usefulness (PU)

PU is the degree to which an individual trusts that accepting/using a specific technology will enhance job performance [58]. Studies on the topic have shown that perceived usefulness affects the intention to use mHealth and exposure detection apps. For instance, Zhang et al. [72] reported a positive PU/intention to use mHealth relationship, and Binyamin and Zafar [73] showed the significant influence of PU on the intention to use an mHealth app. Additionally, PU also significantly influences the intention towards exposure detection apps usage, Alsyouf et al. [4]. Thus, this study posits the following hypothesis.

**Hypothesis** **1** **(H1).** *PU positively influences intention to use exposure detection apps.*

##### Perceived Ease of Use (PEOU)

According to Davis [60], PEOU refers to the degree to which an individual is convinced that using a particular technology will be devoid of effort and difficulty. In this study, PEOU is the belief of a user that using exposure detection apps is mentally and physically easy. Prior related studies concerning the PU/intention to use relationship supported a significant relationship. Binyamin and Zafar [73] reported the significant influence of PEOU on PU in light of mHealth, and Tsai et al. [74] and Lee et al. [75] revealed the positive effect of PEOU on the intention to use an mHealth app. Moreover, in Alsyouf et al. [4], PEOU was reported to significantly affect PU and intention towards exposure detection apps usage. Hence, this study proposes that:

**Hypothesis** **2** **(H2).** *PEOU has a positive influence on PU.*

**Hypothesis** **3** **(H3).** *PEOU positively influences the intention towards using exposure detection apps.*

##### Intention to Use

A top determinant for new technology acceptance is the intention to use, with behavioural intention defined as an individual’s inclination towards performing a particular behaviour [48]. Regarding the use of mHealth in general, and exposure detection apps in particular, intention towards system use is the plan to use the technology. Concerning this, Binyamin and Zafar [73] showed that intention towards mHealth app usage is significantly correlated with its actual use, and similarly, Alsyouf et al. [4] found that the intention to use has a significant effect on actual exposure detection apps usage and thus, this study hypothesises the following:

**Hypothesis** **4** **(H4).** *Intention to use exposure detection apps positively influences its actual use.*

##### Health Anxiety Sensitivity to COVID-19 (CA) and Event-Related Fear (ERF)

Experiencing health anxiety at the time of the pandemic can significantly impact people’s lives in the form of avoidance, stress, and intrusive negative thoughts. Such effects can be linked to negative or ineffective preventative behaviours and actions, as evidenced in past studies such as Gaygisiz et al. [76], Wang et al. [77], Qiu et al. [78], and Alrawad et al. [79].

In the same line of study, Gaygisiz et al. [76] stated that people’s perceptions concerning health-related anxiety are numerous. Thus, studying the factors that influence such anxiety may provide insight into adopting health applications during the pandemic. The COVID-19 pandemic has been found to instigate feelings of fear, death, sickness, helplessness, and stigmatisation. Thus, studies dedicated to its examination may assist in enlightening concerned people about their mental health status, which is needed to help people [80].

Owing to the lack of awareness of probabilities and numbers among people, they may be unable to analyse the risk level by calculating likelihoods and assessing the entire outcomes of alternatives [81]. Thus, they have to depend on intuition and innate feelings to evaluate the risks in events using intuition-based mechanisms with evolutionary importance [82,83]. With instant risks occurring, the use of intuition would assist in avoiding environments that are complicated and dangerous. According to Slovic et al. [83], emotions bring about intuitive risk assessment known as the “risk as feelings” hypothesis, which is the judgments of people concerning dangerous and risky events that depend on a specific person’s emotional feelings, as opposed to the actual likelihood of the probability.

Two emotional feelings that significantly correlate with risks and lead to emergencies are fear and anxiety [84]. Most studies along this line indicate that fear and anxiety enhance public health emergencies, as evidenced by Zika and H1N1 outbreaks several years ago [85,86]. Two major negative valence emotions instigate pessimistic risk evaluations [87,88]. The study of Johnson and Tversky [89] found that people underestimated or overestimated the number of deaths caused by floods/smoking following newspaper reports. Therefore, this study is focused on the specific emotional effects that go beyond the valence on the user’s innovation acceptance, specifically the exposure app, to assist in the surveillance of the COVID-19 pandemic and its confinement.

Lerner and Keltner [90] supported the above contention. They indicated that fear can instigate risk estimates of individuals of negative events such as strokes and terrorist attacks [91]. When they directly face risks, they have a higher likelihood to experience anxiety along with higher innovation acceptance to address such anxiety.

Thus, anxiety can be referred to as a relational construct that responds to a risky event for which a protective decision is adopted. Upon encountering a threatening and risky event, the expectation is that anxiety is aroused as a negative emotional response. In the case of the COVID-19 pandemic, anxiety can bring about the individual’s serious consideration of protective response. If ignored, fear of the COVID-19 pandemic could lead to the non-use of exposure detection apps unless a sense of anxiety is developed. COVID-19 anxiety could thus have a mediating effect on the event-related fear/detection app usage relationship. Thus, the following hypothesis is posited:

**Hypothesis** **5** **(H5).** *The relationship between event-related fear and exposure detection apps usage will be mediated by COVID-19 anxiety.*

##### Social Media Awareness (SMA)

Social media use effects on health behavioural changes are driven by mechanisms that encompass the coverage of the pandemic on social media that increases fear in the public’s hearts and leads them to adopt preventive behaviour [92]. In past literature, mass media usage has been shown to generate positive changes and prevent negative ones regarding health-related behaviours among population members [93]. This may be exemplified by reports of frequent listening to the radio and reading newspapers being related to decreased odds of contracting disease thanks to vaccinations [94]. Similarly, frequent watching of television was found to have a positive relationship with behaviours relating to water, sanitation, and hygiene [95]. Facebook and Twitter, two of the top social media platforms, can provide the public and health institutions with new ways to prevent disease during the pandemic. Social media enables two-way communication between health authorities and the members of the public. The usefulness of social media has also been reported in light of health-promotion interventions such as the prevention of risky sexual behaviours [96], enhanced knowledge and attitudes towards skin cancer [97], and the uptake of maternal influenza vaccine as reported by O’Leary et al. [98], as well as bringing about changes in lifestyles.

According to Lim et al. [99], people may be directed to adopt, support, spread, and share innovative ideas/behaviours through socially mediated social media channels. As a mediating factor, social media encapsulates and supports social norms and contributes to the public’s ability to obtain health information in regards to knowledge, news, and patterns of health behaviour, which can widely proliferate through the effect of social influences on the health behaviours of people and the learning phenomenon through intense observation [100]. Hence, the level to which individuals utilise social media for accessing health information and disease management may significantly affect the results of their health behaviour.

Specifically, past studies have investigated the relationships between specific media access types and information-seeking behaviours. For example, Alhuwail and Abdulsalam [101] revealed that people turn to YouTube to obtain health information instead of Twitter, Snapchat, and Facebook. According to Stawarz et al. [102], people utilise mobile technologies to resolve their specific mental health problems. Thus, based on prior literature, it is essential to study the relationship between social media awareness and exposure detection apps to confine the spread of COVID-19. This study proposes the following hypothesis for testing:

**Hypothesis** **6** **(H6).** *Social media awareness positively influences the intention towards using exposure detection apps.*

##### Perceived Privacy (PP)

Users of COVID-19 apps have time and again raised the issue of privacy. This holds true for media and human rights organisations, who are often working against governments developing such apps to be used by all members of society [103,104,105]. Governments all over the globe have been enforcing social distancing and lockdown measures since the beginning of the pandemic, as suggested by health officials and experts [106,107].

Several measures to this effect lead people to become proactive online. The majority of them employ social media apps frequently to assist in reconnecting with their families and friends [14]. Governments/app developers have often raised privacy issues for social media apps such as YouTube and TikTok. Most app users prefer not to get involved in the debate [108]. Social media and productivity apps often have high privacy and ethical concerns as they can be risky and dangerous to individuals and governments; this may be evidenced by the Australian and U.S. government attempts to prohibit Chinese-owned social media apps such as TikTok and WeChat, claiming that these apps have gathered the users’ personal data, and have built a considerable understanding from the information of those users [109]. Data may also be used unethically, as in the recent U.S. elections, whereby both YouTube and Facebook were used to shift the election outcome to benefit the rival foreign governments [110]. This led to the U.S. government’s decision to ban new downloads for TikTok and WeChat for the citizens’ privacy protection.

Examining the privacy policies and data use agreements that most social media and productivity apps provide can uncover several privacy issues that users lack awareness of, which shows the precedence of data collection over privacy for users. Contrastingly, privacy policies and data usage agreements analysis for most COVID-19 apps indicate that users’ privacy comes first rather than their data [20]. Thus, prior results on the perceived privacy/use of exposure detection apps relationship led this study’s authors to propose the following hypothesis:

**Hypothesis** **7** **(H7).** *Perceived privacy positively influences the intention towards using exposure detection apps.*

##### Trust in Government (TIG) and Social Influence (SI)

The trust element generally mitigates uncertainties and risks in various contexts, Aysan [111], Bélanger and Carter [33], and trust is usually referred to as a psychological state consisting of the intention to accept vulnerability based upon positive expectations of another’s intentions/behaviour [112]. However, trust may also be modified in regards to the actual app usage, either becoming weaker or stronger based on distinct experiences by a user [113]. Additionally, the trust of individuals in their government is another way to mitigate the uncertainties they hold. Most COVID-19 tracing apps have been developed by governments. Trust in the government may mitigate the apps-related fear [114]. Trust in the government is assumed to be stable and not changeable in a short-run period [115,116].

Based on the trust transfer theory, the trust of individuals in a particular area can affect their initial trust in other related areas [117]. Similarly, Lu et al. [118] illustrated that customers’ trust in internet payment affects their trust in mobile payment services. Aligned with this, COVID-19 tracing government-established apps may be supported in their use by supporting the trust of citizens and residents in the government.

In light of the above, the trust of the people in the government refers to the perceptions concerning the integrity and ability of the government agency that provides a service [33]. The conviction of the people that the government’s actions are directed towards their best interests would bring about the belief that government agencies are capable of suitably providing services, strengthening their initial trust in using COVID-19 apps [30]. Recent findings on COVID-19 tracing apps show that trust in the government significantly affects people’s attitudes towards the apps [114,119].

Because of the impossibility that people could test-run the COVID-19 exposure detection apps before their launch or interact with those responsible for their creation, people turn to other options for interactive information collection, such as communicating with peers who are also influenced by the decision as to whether to use the app. In other words, social influence is an active information-seeking method. It is expected to mitigate the uncertainty concerning COVID-19 tracing apps among people. More importantly, social influence refers to the level to which an individual perceives that other individuals important to him are convinced that he should use the new system [32]. Determining the preferences of individuals concerning the social environment would reveal their attitude towards app usage.

Moreover, the primary reason behind general negative attitudes towards COVID-19 tracing apps is the people’s lack of government trust [119]. Social influence can promote protective responses among individuals towards seriously considering the pandemic. Because trust in government could be overlooked, such trust alone may be unable to increase exposure detection app usage unless other alternatives are sought for gathering interactive information through peers who are also in the same predicament (whether or not to use the app). Social influence is thus expected to mediate the trust in the government/exposure detection app use relationship. Therefore, this study proposes the following hypothesis for testing:

**Hypothesis** **8** **(H8).** *Social influence mediates the relationship between trust in government and exposure detection app usage.*

## 2. Materials and Methods

### 2.1. Research Context

Saudi Arabia has not been spared the impact of COVID-19, as with other countries in the world. As a result, the MOH of Saudi Arabia has launched the Tabaud and Tawakkalna apps [4,5]. Specifically, the Tabaud app to be used in smartphones is among the latest efforts made by the Saudi government to fight against and contain the COVID-19 pandemic through Apple/Google exposure notification API. The institution responsible for developing the app is the National Information Center (NIC) of Saudi Data and Artificial Intelligence Authority (SDAIA), in collaboration with the Ministry of Health (MOH).

The app offers three major user services, namely sending notifications of close contact with COVID-19-infected individuals, helping by forwarding their health forms to the MOH for the required medical support based on the case status and progress, and allowing the confirmed infected cases to voluntarily display their test results to those they are in contact with during a 2-week period.

Tabaud is an app that respects users’ privacy. Using it requires no information/location sharing, as it depends on Bluetooth technology to collect and update IDs at random. Users who want to begin the medical testing procedure for the virus would need to forward their name, national or residence ID number, and date of birth.

Tawakkalna is described as the official application launched by Saudi Arabia to prevent the COVID-19 virus from spreading. It was created using the Saudi Data and Artificial Intelligence Authority (SDAIA). Its initial launch was focused on contributing to managing relief efforts through electronic means and enabling a curfew period for government and private sector employees and individuals. This would work towards confining the COVID-19 virus spread in the country. The “Return with Caution” period heralded the launching of several new services in the Tawakkalna app, contributing to achieving “safe return” while making the users’ statuses clear via coloured codes with the highest security and privacy levels.

1 Dark Green Colour Code in Tawakkalna: Immunity from COVID-19

This Colour Code is further divided into three:

(a)   IMMUNE—dark green colour shows the completion of the COVID-19 vaccine.
(b)   IMMUNE BY FIRST DOSE—this shows that the user has received a portion of the vaccine. It is displayed for two weeks following the latest vaccine dose. It continues for another 180 days until the total doses are completed or an infection is detected.
(c)   IMMUNE BY RECOVERY—this shows the recovery of the user from the infection and that they have developed a natural immunity from it lasting 6 months unless another infection arises, or a vaccine is received.

2. Green Colour: No Record of Infection

This shows no infection record of the user or no direct contact with an infected person or shows exposure to an infected person but, when retested, the user has recovered (declared healthy).

3. Orange Colour: Exposed to COVID-19

This shows the user’s exposure to a COVID-19-infected person and the user is allowed to leave the residence but not allowed to go into workplaces/enclosed public places or obtain permits.

4. Brown Colour: Infected by COVID-19

This shows that the user is infected with COVID-19 under the Ministry of Health data.

5. Blue Colour: Arrived from Abroad: Category A Countries

This shows that the user travelled from countries not included in the Ministry of Health list of countries, completed 7 days of self-quarantine, or adhered to the 3 day self-quarantine by testing for the virus 48 h following his arrival to Saudi Arabia, which automatically terminates the self-quarantine upon a negative result. This shows a “No record of infection” health condition.

6. Violet Colour: Arrived from Abroad: Category B Countries

This shows that the user arrived from countries defined under Category B by the Ministry of Health, completed 7 days of self-quarantine, and took a virus test on the 6th day to terminate the self-quarantine with a negative test result. Like the blue colour, a “No record of infection” appears as a health condition.

7. Grey Colour: No Internet Connection

This shows the absence of internet connection on the individual’s device or the non-location of address or the use of a virtual private network (VPN).

The Tabaud and Tawakkalna apps work together, with Tabaud detecting, surveilling, and sending COVID-19 cases to the Tawakkalna app, while the latter clarifies the health status of the user through the colour codes, using the highest security and privacy degrees [3,4].

Saudi public health agencies have attempted to leverage the extensive penetration of the internet at a low cost and the use of smartphones in the Kingdom to contain the COVID-19 spread. Based on the latest reports, the number of active smartphone users has surpassed 33 million in the Kingdom of Saudi Arabia [120]. Saudi public health agencies have forwarded a message to all smartphone users introducing Tabaud and Tawakkalna apps to promote their use. Additionally, these agencies have also published and distributed materials concerning the apps over social and mainstream media to boost the adoption and use of apps among smartphone users and raise their familiarity with the apps.

Concerning the above, the status “immune from COVID-19” in the Tawakkalna app is one of the prerequisites to enter and take part in activities (economic, commercial, cultural, sports, tourism, scientific, social, and recreational). This study shows that 100% of Saudi citizens and residents have downloaded both apps to effectively control the spread of COVID-19. It examines the factors influencing the users’ actual use of the apps.

### 2.2. Sample and Data Collection

This study used a quantitative approach and cross-sectional design to test the proposed model. The government imposed lockdown measures and social distancing made the physical gathering of data impossible. An online survey questionnaire has been used as an instrument for data collection during the lockdown period. This study targeted the smartphone users in Saudi Arabia. Due to the challenge in obtaining a list of smartphone users in the country, the faculty members, employees, and students at King Abdul-Aziz University were surveyed; the university is a place where there is a wide usage of smartphones. Furthermore, the social distance and lockdown imposed by the government made data collection procedures from a representative sample an incredible mission. In view of these limitations, collecting data from the university was the best option for the current study purposes.

After the survey was prepared in Arabic, the main language of probable respondents, an online-based questionnaire through the survey link was forwarded by the university email distribution group to the respondents (faculty, employees, and students) at the university. The survey link was also published and shared on popular social media platforms, including university communities. Data collection was conducted from 15 October to 15 December 2021. Krejcie and Morgan’s [121] table was used to estimate the sample size. By examining sample size criteria established by Krejcie and Morgan [121], it was determined that a sample size of 384 was acceptable for generalising results. In total, 586 participants’ surveys were retrieved. Upon examination, all surveys were complete, indicating no missing data.

The original survey was translated into Arabic, which is the language of potential respondents. The survey content was designed by adopting validated instruments from past literature, with items gauged on a 5-point Likert scale ranging from 1 denoting strongly disagree to 5 denoting strongly agree. The variables’ measurement scales were adopted from relevant articles and the list of measurement items is available in Appendix A.

The respondents’ demographic information analysis results show that most were male, with the descriptive results showing 310 male respondents (52.9%), with the majority of respondents falling into the age category of 18–34 years of age (362 respondents, 61.8%), having bachelor’s degrees (431 respondents, 73.5%), living in the Western Saudi province (431 respondents, 73.5%). The demographic characteristics of the respondents are tabulated in Table 1, including gender, age, residence location, and level of education.

## 3. Results

The developed framework was tested using partial least squares (PLS), with SEM enabling the simultaneous examination of the measurement and structural models [122,123,124]. PLS is also effective when dealing with complicated models, characterised by a hierarchical structure, several indicators, relationships, and constructs [125,126,127,128]. Moreover, PLS also addresses issues brought on by small-sized samples and error terms, with few rigid assumptions of normal data distribution [125,126,129,130]. More specifically, the proposed model was tested using PLS version 3.0 M3. The first step entailed testing the reliability and validity of the measurement model [131,132]. This involved establishing the model’s convergent validity through AVE, indicator reliability, internal consistency, and discriminant validity [124,133].

The values of Cronbach’s alpha (CA), composite reliability (CR), item loadings, and AVE of the constructs are tabulated in Table 2. Based on the table, CA and CR were over the threshold value of 0.70 for the entire constructs, indicating that both internal consistency and suitability of constructs are well-established based on the suggestion of Hair et al. [134], Hair et al. [135]. All factors had reliability of more than 0.40, so they were all acceptable.

Convergent validity, measured through AVE, was more than 0.50, which is the conventional cut-off value. The squared AVE values of the constructs were used to evaluate discriminant validity, and they appeared to be higher than the correlation constructs, and thus they achieved discriminant validity of the constructs (refer to Table 3).

The direct and mediating effects proposed in the model were tested to examine the formulated hypotheses in the structural model. Accordingly, a PLS path algorithm that generated the path coefficients was conducted to evaluate the study significance in a process involving the assessment of the structural model. The bootstrapping procedure was used with a generated 5000 sample [124,130,134,136]. The path coefficient significance was evaluated in two parts: direct effect and mediating effect (refer to Table 4 and Table 5). 

The results tabulated in Table 4 support a significant and positive relationship between citizens’ intention towards exposure detection apps and perceived usefulness (β = 0.231, *t* = 3.730, *p*< 0.01), supporting H1. The same held true for perceived ease of use relationship with perceived usefulness of exposure detection apps (β = 0.667, *t* = 22.232, *p*< 0.01), indicating support of H2, and the positive relationship between perceived ease of use and intention towards exposure detection apps and its use among the Saudi citizens (β = 0.250, *t* = 3.789, *p* < 0.01), indicating support for H3. The relationship between intention towards exposure detection apps and intentions to use was found to significantly and positively impact its actual use (β = 0.526, *t* = 12.897, *p* < 0.01), which means H4 is supported. On the other hand, the impact of social media awareness on exposure detection apps usage was positive but not significant (β = 0.019, *t* = 0.463), and thus, H6 is rejected. Regarding perceived privacy, its impact on exposure detection apps intention towards use was positive and significant (β = 0.295, *t* = 5.273, *p* < 0.01). Thus, H7 is supported.

Moving on to the mediating relationships, for H5 and H8, according to [137], social influence and anxiety mediate the relationship between event-related fear and the use of exposure detection apps (refer to Table 5). Based on their suggestion, mediation exists if there is an insignificant indirect effect and the bootstrapped confidence interval fails to straddle a 0 in-between.

In this study, the bootstrapping analysis on trust in government illustrated a significant indirect effect at (β = 0.061) with *t*-value = 4.306, with the indirect effect 95% of exposure detection apps usage (LL = 0.038, UL = 0.085) illustrating the absence of straddling a 0 in-between, as explained by [137]. The same held true for event-related fear and its significant indirect effect (β = 0.079) with *t*-value = 3.966, with indirect effect 95% of bootstrapped exposure detection apps use (LL = 0.047, UL = 0.114) and absence of 0 in-between straddle.

The above results show that social influence and COVID-19 anxiety have a mediating role in the relationship between trust in government and exposure detection app use and event-related fear and exposure detection apps use, supporting Hypotheses 5 and 8.

## 4. Discussion

This study validated the effectiveness and benefit of TAM in predicting exposure detection apps use for tracing cases and individuals who have recently come into close contact with positive COVID-19 cases, assisting in breaking the infection chain. The assumptions of TAM were supported with additional variables, providing the model with higher predictive strength. Notably, the model explained 0.364 in COVID-19 anxiety, 0.477 in exposure detection apps intention, and 0.459 in exposure detection apps usage. Moreover, social influence displayed a low predictive strength of 0.101.

Based on the results, perceived usefulness significantly predicted exposure detection apps intention (*p* < 0.01), which is aligned with past studies dedicated to mHealth such as Binyamin and Zafar [73], Sezgin et al. [138], and Zhang et al. [72]; and is aligned with exposure detection apps such as Alsyouf et al. [4]—studies reported that PU is a top driver of user’s behavioural intention to use mHealth types. Thus, if citizens are convinced of the usefulness of exposure detection apps in safeguarding their health from COVID-19 infection, they will have a high usage rate.

In H2, the relationship between ease of use and perceived usefulness was tested. A significant relationship was found (*p* < 0.01), similar to past studies by Binyamin and Zafar [73], Li et al. [75], and Tsai et al. [74] in the mHealth context, and by Alsyouf et al. [4] in the exposure detection apps context. This shows that the perception of ease in using exposure detection apps may lead to the belief that they are useful. As such, users who find the app easy to use would use it more often, which would support their perception of its usefulness and importance to their lives.

Moreover, a significant relationship was found between PEOU and exposure detection apps intention (*p* < 0.01) in H3. The same finding was found in past studies by Binyamin and Zafar [73], Deng et al. [139], and Zhu et al. [140] in mHealth, and by Alsyouf et al. [4] in exposure detection apps. The results show that perceived ease of use among citizens regarding exposure detection apps could lead to their effective intention to use them and actual use of the apps. This result may be attributed to the crucial significance of PEOU of mHealth among citizens. In past studies, consumers’ acceptance of health informatics applications was revealed to be distinct from how health professionals accept them [65,69]. This was due to consumers’ lack of self-efficacy and negative feelings regarding usability, making consumers more likely to encounter challenges in using health informatics applications. In other words, it is necessary to assist citizens’ acceptance of the app.

In the fourth hypothesis (H4), intention to use exposure detection apps was found to be a major indicator of its actual use, as a positive influence was found (*p* < 0.01). The significant relationship of the two factors was also supported in past studies (e.g., [4,73,141,142]). This finding indicates that the users’ behavioural intention is a good indicator of their acceptance and use of new technology. In other words, citizens’ intention towards using exposure detection apps predicts their actual acceptance and use of the same juxtaposed against prior studies in the mHealth context.

The influence of social media awareness on exposure detection apps intention to use was positive but insignificant among Saudi citizens, indicating that social media messages disseminating the information may have no effects on the use because the use of the apps has been made mandatory. The Saudi government adopted a paternalistic strategy in their emergency response, thereby promoting the common good over the individual’s right to autonomy [143]. In a paternalistic strategy, everyone’s freedom is restricted to protect everyone’s best interests, giving the rights of society priority over individual rights. Moreover, individuals have the right to health, including protection from and prevention of contracting diseases, to ensure long-term interests’ precedence over short-term ones.

In Saudi Arabia, public health policies are directed towards population-level health outcomes rather than the individuals’ rights and interests, making it mandatory for citizens to use exposure detection apps. For instance, to physically access any economic, commercial, cultural, sports, tourism, scientific, social, or recreational activity in the Kingdom, the status, “Immune from COVID-19” must be present. To this end, social media awareness has no significant effect on the use of exposure detection apps among citizens.

This result may also be attributed to increased social media use during the pandemic. An increasing number of people engage and connect with others online, boosting their sharing of information [144,145]. At the same time, social media has a crucial role in spreading misinformation and sensationalism concerning COVID-19, thus, emotionally charging users and attracting their attention [146]. Hence, social media trust as a dependable COVID-19 information source is not as effective; it only has a minimal effect on intention towards exposure detection apps usage and it has a vital role in spreading misinformation and sensationalism concerning COVID-19.

The results of H7 show that perceived privacy predicted that exposure detection apps intention to use was (*p* < 0.01), which is similar to that reported by past studies on mHealth (e.g., [147,148]). This finding reveals the importance of perceived privacy on the behavioural intention of the user to use mHealth and its types. Thus, perceived privacy contributes to the adoption of exposure detection app intention. If users perceive that their privacy is valued by the app, not their data, they will be more inclined to use it. In this context, the Tabaud app is privacy-focused. Using it requires no information or location sharing; it depends on Bluetooth to obtain IDs and updates them randomly. A user who needs any medical procedure or testing for the virus can avail the information provided by the Ministry of Health regulations. They require personal information (name, national or residence ID number, and date of birth).

Added to the above, social influence was revealed to have a mediating role in the relationship between trust in government and exposure detection apps use (*p* < 0.01). This finding is a new contribution to the literature that shows a significant and direct positive effect of trust in government on exposure detection apps use and on social influence. Social influence was also found to significantly affect exposure detection app usage. In this regard, no empirical findings have been documented on the mediating impact of social influence on the trust in government/exposure detection apps usage relationship.

In the same way, the mediating role of COVID-19 anxiety on the relationship between event-related fear and exposure detection apps usage was supported (*p* < 0.01), contributing another new finding to the literature. This result shows that event-related fear significantly and directly affects exposure detection apps in a positive direction. This held the same for the direct effect of event-related fear on COVID-19 anxiety, as with anxiety’s direct effect on using exposure detection apps. Nevertheless, empirical studies on the mediating effects of COVID-19 anxiety on the event-related fear/exposure detection apps usage relationship are still lacking.

In sum, social influence and COVID-19 anxiety significantly mediated the relationship between trust in government/exposure detection apps use and COVID-19 anxiety/exposure detection apps usage, supporting H5 and H8. The statistical findings supported the conceptual model’s predictive validity, thereby validating the premise that trust in government and event-related fear caused by COVID-19 can enhance the use of exposure detection apps.

## 5. Conclusions

This study minimises the literature gap concerning the relationships between constructs and acceptance of exposure detection apps, including social media awareness, perceived privacy, with social influence and trust in government as new exogenous predictors of TAM among Saudi citizens’ use of the apps. According to Jaber et al. [149], the major public health challenges for technologies in the current times are related to COVID-19 health monitoring and management.

Based on the study’s findings, there are several implications for management. The government of Saudi Arabia is concerned with the public’s safety compliance behaviours, including social distancing, wearing masks, and hygiene [41]. In this regard, the exposure detection apps provide solutions for surveillance of COVID-19 cases through e-input, transmission, and data retrieval from local and remote locations. Consequently, the detection and reporting of any potential case can be traced. Enabling tracing is smartphone communication technology that has become ubiquitous in daily life in all sectors. Policymakers in healthcare throughout the globe make use of mHealth to confine the pandemic and control the health crisis while enhancing health services even in remote areas with low resources. Which will lead to improve Patients safety too [150].

The COVID-19 pandemic opens up avenues for extending, integrating, and theoretically testing technology acceptance models. Such replications, applications, and integrations to TAM specifically contribute to understanding current technology adoption. Furthermore, the study is directed towards explaining the influence of social media awareness, perceived privacy, social influence, and trust in government on the perceptions towards using exposure detection apps during the pandemic. These attitudes and behaviours are crucial for public health officials, technology developers, and experts in order to design better apps, implement better techniques, and better protect personal information. Consequently, app updates can be released, addressing the public worries based on their feedback and ultimately enhancing the exposure detection apps adoption and usage rates.

### Limitations and Future Research

This study has limitations, the first being the application of the study conclusions to one location and time. The study conducted a cross-sectional survey that needs to account for the differences among the underlying associations throughout divisions, locations, contexts, and countries. Their meanings may dissipate as time passes. Future studies could use a longitudinal design. Second, data were collected through university email distribution groups from one of the biggest universities in Saudi Arabia, limiting the generalisability of the results. Collecting data online during the lockdown period limited the collection of more accurate and representative data for the study. Future research should consider different settings and include bigger samples representing the Saudi context better.

In addition, other methods of data collection may be used in the future; further research may include comparative studies or assessment of pre-adoption and post-adoption behaviour in regards to mobile health applications. Moreover, qualitative research could be used to acquire life experiences that are pertinent to the positional analysis adopted here by eliciting narrative analysis or explanation phenomenology approaches.

Third, the present study does not consider all external variables that impact technology acceptance; more research is required through extending TAM with other external variables, such as technology self-efficacy, satisfaction, and quality factors (services quality, system quality, and information quality). In addition, demographic parameters such as age and gender could be addressed.

Fourth, the present study focuses purely on TAM. In the future, it would be interesting to combine TAM with other theories. Ultimately, it may be necessary to re-examine the findings of this study in other contexts in the future. Finally, future studies could address the impact of the adopted paternalistic strategy by governments on the adoption of tracing apps to fight COVID-19 pandemic spreads.

## Figures and Tables

**Table 1:** Demographic characteristics of the respondents.

Demographic Characteristics	Category	N	%
Gender	Male	310	52.9
	Female	276	47.1
	Total	586	100
Age	17 years old and younger	5	0.9
	18–34 years old	362	61.8
	35–44 years old	86	14.7
	45–54 years old	78	13.3
	55–64 years old	45	7.7
	65 years and over	10	1.7
	Total	586	100
Education level	High school degree and below	131	22.4
	Diploma certificate	28	4.8
	Bachelor’s degree	321	54.8
	Master’s degree	58	9.9
	PhD holders	48	8.2
	Total	586	100
Province	Western province	431	73.5
	Eastern province	66	11.3
	Southern province	0	0.0
	Northern province	68	11.6
	Middle province	21	3.6
	Total	586	100

**Table 2:** Item loading, Cronbach’s alpha, composite reliability, average variance extracted.

Construct	Measurement Items	Loadings	Cronbach’s Alpha	Composite Reliability	Average Variance Extracted (AVE)
COVID-19 Anxiety (CA)	CA1	0.781	0.701	0.808	0.515
	CA7	0.784			
	CA8	0.624			
	CA9	0.669			
Exposure Detection Apps Intention (EDAI)	EDAI1	0.951	0.928	0.954	0.874
	EDAI2	0.905			
	EDAI3	0.948			
Exposure Detection Apps Usage (EDAU)	EDAU1	0.855	0.891	0.933	0.823
	EDAU2	0.945			
	EDAU3	0.919			
Event-Related Fear (ERF)	ERF1	0.925	0.912	0.944	0.850
	ERF2	0.932			
	ERF3	0.908			
Perceived Ease of Use (PEOU)	PEOU1	0.894	0.871	0.911	0.721
	PEOU2	0.876			
	PEOU3	0.858			
	PEOU4	0.762			
Perceived privacy (PP)	PP1	0.822	0.926	0.942	0.732
	PP2	0.888			
	PP3	0.909			
	PP4	0.861			
	PP5	0.891			
	PP6	0.754			
Perceived Usefulness (PU)	PU1	0.917	0.939	0.956	0.845
	PU2	0.933			
	PU3	0.921			
	PU4	0.906			
Social Influence (SI)	SI1	0.921	0.917	0.948	0.858
	SI2	0.937			
	SI3	0.921			
Social Media Awareness (SMA)	SMA1	0.664	0.823	0.872	0.578
	SMA2	0.802			
	SMA3	0.806			
	SMA4	0.721			
	SMA5	0.799			
Trust in Government (TIG)	TIG1	0.886	0.867	0.917	0.787
	TIG2	0.896			
	TIG3	0.879			

**Table 3:** Discriminant validity of the constructs.

	CA	EDAI	EDAU	ERF	PEOU	PP	PU	SI	SMA	TIG
CA	**0.718**									
EDAI	0.23	**0.935**								
EDAU	0.294	0.637	**0.907**							
ERF	0.603	0.159	0.23	**0.922**						
PEOU	0.168	0.614	0.488	0.11	**0.849**					
PP	0.202	0.613	0.517	0.152	0.683	**0.856**				
PU	0.211	0.586	0.464	0.184	0.667	0.603	**0.919**			
SI	0.214	0.419	0.442	0.244	0.463	0.428	0.63	**0.926**		
SMA	0.161	0.356	0.327	0.207	0.413	0.365	0.545	0.521	**0.761**	
TIG	0.252	0.511	0.372	0.099	0.465	0.554	0.458	0.318	0.312	**0.887**

Note: CA: COVID-19 anxiety, Exposure Detection Apps Intention: EDAI, Exposure Detection Apps Usage: EDAU, ERF: Event-Related Fear, PEOU: Perceived Ease of Use, Perceived Privacy: PP, PU: Perceived Usefulness, Social Influence: SI, Social Media Awareness: SMA, and Trust in Government: TIG.

**Table 4:** The assessment of the structural model.

NO	Hypothesis	Beta	Sample Mean (M)	Standard Deviation (STDEV)	*t*-Statistics	*p*-Value	Sig.	Decision
H1	PU -> EDAI	0.231	0.231	0.062	3.730	0.000	Sig.	Supported ***
H2	PEOU -> PU	0.667	0.668	0.03	22.232	0.000	Sig.	Supported ***
H3	PEOU -> EDAI	0.250	0.245	0.066	3.789	0.000	Sig.	Supported ***
H4	EDAI -> EDAU	0.526	0.527	0.041	12.897	0.000	Sig.	Supported ***
H6	SMA -> EDAI	0.019	0.021	0.041	0.463	0.322	Not sig.	Not Supported
H7	PP -> EDAI	0.295	0.299	0.056	5.273	0.000	Sig.	Supported ***

Note: *t*-values > 1.645 * (*p* < 0.05); *t*-values > 1.96 ** (*p* < 0.02); and *t*-values > 2.33 *** (*p* < 0.01); one-tailed test. SE = Standard Error, LL = Lower Limit, and UL = Upper Limit.

**Table 5:** Summary of mediation results.

					Bootstrapped Confidence Interval		
No	Hypothesis	Indirect Effect (Beta)	SE	*t* Value	5% LL	95% UL	Decision
H5	TIG- > SI- > EDAU	0.061	0.014	4.306	0.038	0.085	Supported ***
H8	ERF- > CA- > EDAU	0.079	0.02	3.966	0.047	0.114	Supported ***

Note: *t*-values > 1.645 * (*p* < 0.05); *t*-values > 1.96 ** (*p* < 0.02); and *t*-values > 2.33 *** (*p* < 0.01); one-tailed test. SE = Standard Error, LL = Lower Limit, and UL = Upper Limit.

## Data Availability

The data presented in this study are available on request from the corresponding author.

## Appendix A

**Table A1:** Variables with measurement items factors.

COVID-19 Anxiety	Items	Reference
CA1	To what extent are you concerned about the COVID-19 pandemic?	[4,151]
CA2	To what extent do you believe that COVID-19 could become a “pandemic” in Saudi Arabia?	
CA3	How likely is it that you could become infected with the COVID-19 pandemic?	
CA4	How likely it is that someone you know could become infected with the COVID-19 pandemic?	
CA5	How quickly do you believe contamination from the COVID-19 pandemic is spreading in Saudi Arabia?	
CA6	If you did become infected with the COVID-19 pandemic, to what extent are you concerned that you will be severely ill?	
CA7	To what extent has the threat of the COVID-19 pandemic influenced your decisions to be around people?	
CA8	To what extent has the threat of the COVID-19 pandemic influenced your travel plans?	
CA9	To what extent has the threat of the COVID-19 pandemic influenced your use of safety behaviours (e.g., hand sanitiser)?	
**Event-Related Fear**	**Items**	**Reference**
ERF1	The current COVID-19 pandemic makes me feel afraid.	[4,152]
ERF2	The current COVID-19 pandemic makes me feel anxious.	
ERF3	“When I think of The current COVID-19 pandemic, I get very scared about what might happen to me”	
**Exposure detection App Usage**	**Items**	**Reference**
EDAU 1	I downloaded the Exposure detection App on my device during the COVID-19 pandemic.	[4,8]
EDAU 2	Currently using the Exposure detection App during the outbreak of the Corona Virus (COVID-19) pandemic.	
EDAU 3	Use the Exposure detection App frequently during the outbreak of the Corona Virus (COVID-19) pandemic.	
**Perceived Usefulness**	**Items**	**Reference**
PU1	Using the Exposure detection App is useful to protect me from the COVID-19 pandemic.	[4,60]
PU2	Using the Exposure detection App increases my attention to the COVID-19 pandemic.	
PU3	Using the Exposure detection App helps me reduce the time it takes to identify infected cases in contact with me	
PU4	The use of the Exposure detection App enhances the efficiency of epidemiological surveillance to isolate people in contact with infected cases during the COVID-19 pandemic.	
**Perceived Ease of Use**	**Items**	**Reference**
PEOU1	I feel that the Exposure detection App is easy to use.	[4,60]
PEOU2	I feel that the Exposure detection App is convenient.	
PEOU3	Getting the information that I want from the Exposure detection App is easy.	
PEOU4	The exposure detection App requires no training.	
**Exposure detection App Intention**	**Items**	**Reference**
EDAI 1	I intend to continue using the Exposure detection App during the COVID-19 pandemic outbreak.	[4,8]
EDAI 2	I will always try to use the Exposure detection App during the COVID-19 pandemic outbreak.	
EDAI 3	I plan to continue to use the Exposure detection App during the COVID-19 pandemic outbreak.	
**Social influence**	**Items**	**Reference**
SI1	People who are important to me think that I should use the Exposure detection App during the COVID-19 pandemic.	[8,31]
SI2	People who influence my behaviour think that I should use the Exposure detection App during the COVID-19 pandemic.	
SI3	People whose opinions are valuable the most will prefer that I use the Exposure detection App during the COVID-19 pandemic.	
**Trust in Government**	**Items**	**Reference**
TIG1	When making important decisions about health regulation during the COVID-19 pandemic, the government is concerned about the welfare of people like me.	[153,154]
TIG2	If I were to have health problems during the COVID-19 pandemic, governmental agencies are available to offer me assistance, support and healthcare services.	
TIG3	Those who make decisions about health regulation in this country during the COVID-19 pandemic seem to understand the needs of people like me.	
TIG4	I am comfortable relying on the government to meet its obligations during the COVID-19 pandemic. Dropped.	
**Perceived privacy**	**Items**	**Reference**
PP1	I would feel safe when I send personal information via the Exposure detection App.	[155]
PP2	I think the Exposure detection App has a high commitment to ensuring the privacy of its users.	
PP3	I think the Exposure detection App complies with the personal data protection laws.	
PP4	In my opinion, the Exposure detection App only collects the personal data of users which will only be required for its activity to detect Coronavirus infected cases.	
PP5	In my opinion, the Exposure detection App respects the privacy rights of users when obtaining personal information.	
PP6	In my opinion, My personal data would not be shared with other institutions without my consent if I used the Exposure detection App.	
**Social media awareness**	**Items**	**Reference**
SMA1	Facebook increases my knowledge and awareness about how to use the Exposure detection App to prevent the COVID-19 epidemic from spreading.	[156]
SMA2	Instagram increases my knowledge and awareness about how to use the Exposure detection App to prevent the COVID-19 epidemic from spreading.	
SMA3	Twitter increases my knowledge and awareness about how to use the Exposure detection App to prevent the COVID-19 epidemic from spreading.	
SMA4	Whats App increases my knowledge and awareness about how to use the Exposure detection App to prevent the COVID-19 epidemic from spreading.	
SMA5	YouTube increases my knowledge and awareness about how to use the Exposure detection App to prevent the COVID-19 epidemic from spreading.	

## References

[1] Lutfi A., Saad M., Almaiah M.A., Alsaad A., Al-Khasawneh A., Alrawad M., Alsyouf A., Al-Khasawneh A.L. Actual Use of Mobile Learning Technologies during Social Distancing Circumstances: Case Study of King Faisal University Students. Sustainability.

[2] Almaiah M.A., Hajjej F., Lutfi A., Al-Khasawneh A., Alkhdour T., Almomani O., Shehab R. (2022). A Conceptual Framework for Determining Quality Requirements for Mobile Learning Applications Using Delphi Method. Electronics.

[3] Almaiah M.A., Hajjej F., Lutfi A., Al-Khasawneh A., Shehab R., Al-Otaibi S., Alrawad M. (2022). Explaining the Factors Affecting Students’ Attitudes to Using Online Learning (Madrasati Platform) during COVID-19. Electronics.

[4] Alsyouf A., Masa’deh R.E., Albugami M., Al-Bsheish M., Lutfi A., Alsubahi N. (2021). Risk of fear and anxiety in utilising health app surveillance due to COVID-19: Gender differences analysis. Risks.

[5] Alsyouf A. (2020). Mobile Health for COVID-19 Pandemic Surveillance in Developing Countries: The case of Saudi Arabia. Solid State Technol..

[6] Statista (2022). Adoption of Government Endorsed COVID-19 Contact Tracing Apps in Selected Countries as of July 2020. https://www.statista.com/statistics/1134669/share-populations-adopted-covid-contact-tracing-apps-countries/.

[7] Venkatesh V., Thong J.Y., Xu X. (2016). Unified theory of acceptance and use of technology: A synthesis and the road ahead. J. Assoc. Inf. Syst..

[8] Venkatesh V., Thong J.Y., Xu X. (2012). Consumer acceptance and use of information technology: Extending the unified theory of acceptance and use of technology. MIS Q..

[9] Al-Syouf A.M. (2017). Personality, Top Management Support, Continuance Intention to Use Electronic Health Record System among Nurses in Jordan. Ph.D. Thesis.

[10] Scherer R., Siddiq F., Tondeur J. (2019). The technology acceptance model (TAM): A meta-analytic structural equation modeling approach to explaining teachers’ adoption of digital technology in education. Comput. Educ..

[11] Matos M., McEwan K., Kanovský M., Halamová J., Steindl S.R., Ferreira N., Mariana L., Daniel R., Kenichi A., Margarita G.M. (2022). Compassion protects mental health and social safeness during the COVID-19 pandemic across 21 countries. Mindfulness.

[12] Chi C.G., Ekinci Y., Ramkissoon H., Thorpe A. (2022). Evolving effects of COVID-19 safety precaution expectations, risk avoidance, and socio-demographics factors on customer hesitation toward patronizing restaurants and hotels. J. Hosp. Mark. Manag..

[13] Rodrigues P.C., Borges A.P. (2022). Anxiety during the pandemic: The perceptions of health importance, health knowledge, and health consciousness. Handbook of Research on Interdisciplinary Perspectives on the Threats and Impacts of Pandemics.

[14] Nabity-Grover T., Cheung C.M., Thatcher J.B. (2020). Inside out and outside in: How the COVID-19 pandemic affects self-disclosure on social media. Int. J. Inf. Manag..

[15] Kurniasari F., Bilgin M.H., Danis H., Demir E., Vale S. (2021). Implementation of productivity apps to increase financial inclusion in peer-to-peer lending platform. Eurasian Economic Perspectives.

[16] Cho H., Ippolito D., Yu Y.W. Contact Tracing Mobile Apps for COVID-19: Privacy Considerations and Related Trade-Offs. https://arxiv.org/pdf/2003.11511.pdf.

[17] Lauer S.A., Grantz K.H., Bi Q., Jones F.K., Zheng Q., Meredith H.R., Azman A.S., Reich N.G., Lessler J. (2020). The incubation period of coronavirus disease 2019. (COVID-19) from publicly reported confirmed cases: Estimation and application. Ann. Intern. Med..

[18] Watkins J. (2020). Preventing a COVID-19 pandemic. BMJ.

[19] Abeler J.B. (2021). COVID-19 contact tracing and data protection can go together. JMIR Mhealth Uhealth.

[20] Haggag O., Haggag S., Grundy J., Abdelrazek M. COVID-19 vs social media apps: Does privacy really matter?. Proceedings of the 2021 IEEE/ACM 43rd International Conference on Software Engineering: Software Engineering in Society (ICSE-SEIS).

[21] Chen H.T. (2018). Revisiting the privacy paradox on social media with an extended privacy calculus model: The effect of privacy concerns, privacy self-efficacy, and social capital on privacy management. Am. Behav. Sci..

[22] Ferretti L., Wymant C., Kendall M., Zhao L., Nurtay A. (2020). Quantifying SARS-CoV-2 transmission suggests epidemic control with digital contact tracing. Science.

[23] Wnuk A., Oleksy T., Maison D. (2020). The acceptance of COVID-19 tracking technologies: The role of perceived threat, lack of control, and ideological beliefs. PLoS ONE.

[24] Wong W. (2020). Technology Threatens Human Rights in the Coronavirus Fight. https://theconversation.com/technology-threatens-human-rights-in-the-coronavirus-fight-136159.

[25] Agamben G. (2022). State of Exception.

[26] Scheppele K.L. (2003). Law in a time of emergency: States of exception and the temptations of 9/11. Univ. Pennslyvania. J. Const. Law.

[27] Trang S., Trenz M., Weiger W.H., Tarafdar M., Cheung C.M. (2020). One app to trace them all? Examining app specifications for mass acceptance of contact-tracing apps. Eur. J. Inf. Syst..

[28] Fenton N.E., McLachlan S., Lucas P., Dube K., Hitman G.A., Osman M., Kyrimi E., Neil M. (2021). A Bayesian network model for personalised COVID19 risk assessment and contact tracing. MedRxiv.

[29] Parker M.J., Fraser C., Abeler-Dörner L., Bonsall D. (2020). Ethics of instantaneous contact tracing using mobile phone apps in the control of the COVID-19 pandemic. J. Med. Ethics.

[30] Dehmel S., Kenning P., Wagner G.G., Liedtke C., Micklitz H.W., Specht-Riemenschneider L. (2020). Die Wirksamkeit der Corona-Warn- App Wird Sich nur im Praxistest Zeigen Der Datenschutz Ist nur Eine von Vielen Herausforderungen POLICY BRIEF.

[31] Alsyouf A., Ishak A.K. (2018). Understanding EHRs continuance intention to use from the perspectives of UTAUT: Practice environment moderating effect and top management support as predictor variables. Int. J. Electron. Healthc..

[32] Venkatesh V., Morris M.G., Davis G.B., Davis F.D. (2003). User acceptance of information technology: Toward a unified view. MIS Q..

[33] Bélanger F., Carter L. (2008). Trust and risk in e-government adoption. J. Strateg. Inf. Syst..

[34] McKnight C., Choudhury V., Kacmar C. (2002). Developing and validating trust measures for e-commerce: An integrative typology. Inf. Syst. Res. INFORMS.

[35] Al-Hanawi M.K., Angawi K., Alshareef N., Qattan A., Helmy H., Abudawood Y., Alqurashi M., Kattan W.M., Kadasah N.A., Chirwa G.C. (2020). Knowledge, attitude and practice toward COVID-19 among the public in the Kingdom of Saudi Arabia: A cross-sectional study. Front. Public Health.

[36] Lutfi A., Al-Khasawneh A.L., Almaiah M.A., Alsyouf A., Alrawad M. (2022). Business Sustainability of Small and Medium Enterprises during the COVID-19 Pandemic: The Role of AIS Implementation. Sustainability.

[37] Al-Rawajfah O.M., Al-Mugeed K.A., Alaloul F., Al-Rajaibi H.M., Omari O.A. (2021). COVID-19 knowledge, attitude, and precautionary practices among health professional students in Oman. Nurse Educ. Pract..

[38] Páez-Osuna F., Valencia-Castañeda G., Rebolledo U.A. (2022). The link between COVID-19 mortality and PM2. 5 emissions in rural and medium-size municipalities considering population density, dust events, and wind speed. Chemosphere.

[39] Xu X., Chen P., Wang J., Feng J., Zhou H., Li X., Zhong W., Hao P. (2020). Evolution of the novel coronavirus from the ongoing Wuhan outbreak and modeling of its spike protein for risk of human transmission. Sci. China Life Sci..

[40] WorldOmeter (2022). COVID-19 Coronavirus Pandemic. https://www.worldometers.info/coronavirus/.

[41] Al-Bsheish M., Jarrar M., Scarbrough A. (2021). A Public Safety Compliance Model of Safety Behaviors in the Age of the COVID-19 Pandemic. INQUIRY J. Health Care Organ. Provis. Financ..

[42] Garousi V., Cutting D., Felderer M. (2022). Mining user reviews of COVID contact-tracing apps: An exploratory analysis of nine European apps. J. Syst. Softw..

[43] Elkhodr M., Mubin O., Iftikhar Z., Masood M., Alsinglawi B., Shahid S., Alnajjar F. (2021). Technology, privacy, and user opinions of COVID-19 mobile apps for contact tracing: Systematic search and content analysis. J. Med. Internet Res..

[44] Hinch R., Probert W., Nurtay A., Kendall M., Wymant C., Hall M., Fraser C. (2020). Effective Configurations of a Digital Contact Tracing App: A Report to NHSX. https://cdn.theconversation.com/static_files/files/1009/Report_-_Effective_App_Configurations.pdf?1587531217.

[45] Breil B., Salewski C., Apolinário-Hagen J. (2022). Comparing the acceptance of mobile hypertension apps for disease management among patients versus clinical use among physicians: Cross-sectional survey. JMIR Cardio.

[46] Yang M., Jiang J., Kiang M., Yuan F. (2021). Re-examining the impact of multidimensional trust on patients’ online medical consultation service continuance decision. Inf. Syst. Front..

[47] Hassandoust F., Subasinghage M., Johnston A.C. (2022). A neo-institutional perspective on the establishment of information security knowledge sharing practices. Inf. Manag..

[48] Fishbein M., Ajzen I. (1977). Belief, Attitude, Intention, and Behavior: An Introduction to Theory and Research.

[49] Ajzen I. (1991). The Theory of Planned Behavior. Organ. Behav. Hum. Decis. Processes.

[50] Klobas J.E., McGill T., Wang X. (2019). How perceived security risk affects intention to use smart home devices: A reasoned action explanation. Comput. Secur..

[51] Procter L., Angus D.J., Blaszczynski A., Gainsbury S.M. (2019). Understanding use of consumer protection tools among Internet gambling customers: Utility of the Theory of Planned Behavior and Theory of Reasoned Action. Addict. Behav..

[52] Meyer-Waarden L., Cloarec J. (2022). “Baby, you can drive my car”: Psychological antecedents that drive consumers’ adoption of AI-powered autonomous vehicles. Technovation.

[53] Hoque R., Sorwar G. (2017). Understanding factors influencing the adoption of mHealth by the elderly: An extension of the UTAUT model. Int. J. Med. Inform..

[54] Lutfi A. (2022). Factors Influencing the Continuance Intention to Use Accounting Information System in Jordanian SMEs from the Perspectives of UTAUT: Top Management Support and Self-Efficacy as Predictor Factors. Economies.

[55] Lutfi A. (2021). Understanding cloud based enterprise resource planning adoption among smes in jordan. J. Theor. Appl. Inf. Technol..

[56] Almaiah M.A., Al-Otaibi S., Lutfi A., Almomani O., Awajan A., Alsaaidah A., Alrawad M., Awad A.B. (2022). Employing the TAM Model to Investigate the Readiness of M-Learning System Usage Using SEM Technique. Electronics.

[57] Almaiah M.A., Ayouni S., Hajjej F., Lutfi A., Almomani O., Awad A.B. (2022). Smart Mobile Learning Success Model for Higher Educational Institutions in the Context of the COVID-19 Pandemic. Electronics.

[58] Davis F.D. (1985). A Technology Acceptance Model for Empirically Testing New End-User Information Systems: Theory and Results. Ph.D. Thesis.

[59] Davis F.D., Bagozzi R.P., Warshaw P.R. (1989). User acceptance of computer technology: A comparison of two theoretical models. Manag. Sci..

[60] Davis F. (1989). Perceived usefulness, perceived ease of use, and user acceptance of information technology. MIS Q..

[61] Lindberg R.S., De Troyer O. (2020). Towards a reference model of guidelines for the elderly based on technology adoption factors. Proceedings of the 6th EAI International Conference on Smart Objects and Technologies for Social Good.

[62] Baptista G., Oliveira T. (2016). A weight and a meta-analysis on mobile banking acceptance research. Comput. Hum. Behav..

[63] Chen S., Schreurs L., Pabian S.V. (2019). Daredevils on social media: A comprehensive approach toward risky selfie behavior among adolescents. New Media Soc..

[64] Dawson C.H., Mackrill J.B., Cain R. (2017). Assessing user acceptance towards automated and conventional sink use for hand decontamination using the technology acceptance model. Ergonomics.

[65] Tao D., Wang T., Wang T., Zhang T., Zhang X., Qu X. (2020). A systematic review and meta-analysis of user acceptance of consumer-oriented health information technologies. Comput. Hum. Behav..

[66] Tao D., Shao F., Wang H., Yan M., Qu X. (2020). Integrating usability and social cognitive theories with the technology acceptance model to understand young users’ acceptance of a health information portal. Health Inform. J..

[67] Hsu H.-H., Wu Y.-H. (2017). Investigation of the effects of a nursing information system by using the technology acceptance model. CIN Comput. Inform. Nurs..

[68] Alsyouf A. (2021). Self-efficacy and personal innovativeness influence on nurses beliefs about EHRS usage in Saudi Arabia: Conceptual model. Int. J. Manag. (IJM).

[69] Hwang Y., Al-Arabiat M., Shin D.H. (2016). Understanding technology acceptance in a mandatory environment: A literature review. Inf. Dev..

[70] Mathieson K., Peacock E., Chin W. (2001). Extending the technology acceptance model: The influence of perceived user resources. ACM SIGMIS Database DATABASE adv. Inf. Syst..

[71] Venkatesh V., Davis F.D. (2020). A theoretical extension of the technology acceptance model: Four longitudinal field studies. Manag. Sci..

[72] Zhang X., Han X., Dang Y., Meng F., Guo X., Lin J. (2017). User acceptance of mobile health services from users’ perspectives: The role of self-efficacy and response-efficacy in technology acceptance. Inform. Health Soc. Care.

[73] Binyamin S.S., Zafar B.A. (2021). Proposing a mobile apps acceptance model for users in the health area: A systematic literature review and meta-analysis. Health Inform. J..

[74] Tsai T.H., Lin W.Y., Chang Y.S., Chang P.C., Lee M.Y. (2020). Technology anxiety and resistance to change behavioral study of a wearable cardiac warming system using an extended TAM for older adults. PLoS ONE.

[75] Li J., Ma Q., Chan A.H., Man S.S. (2019). Health monitoring through wearable technologies for older adults: Smart wearables acceptance model. Appl. Ergon..

[76] Gaygisiz Ü., Gaygisiz E., Özkan T., Lajunen T. (2012). Individual differences in behavioral reactions to H1N1 during a later stage of the epidemic. J. Infect. Public Health.

[77] Wang C., Pan R., Wan X., Tan Y., Xu L., Ho C.S., Ho R.C. (2020). Immediate psychological responses and associated factors during the initial stage of the 2019 coronavirus disease (COVID-19) epidemic among the general population in China. Int. J. Environ. Res. Public Health.

[78] Qiu J., Shen B., Zhao M., Wang Z.X., Xu Y. (2020). A nationwide survey of psychological distress among Chinese people in the COVID-19 epidemic: Implications and policy recommendations. Gen. Psychiatry.

[79] Alrawad M., Lutfi A., Alyatama S., Elshaer I.A., Almaiah M.A. (2022). Perception of Occupational and Environmental Risks and Hazards among Mineworkers: A Psychometric Paradigm Approach. Int. J. Environ. Res. Public Health.

[80] Alkhamees A.A., Alrashed S., Alzunaydi A., Almohimeed A., Aljohani M. (2020). The psychological impact of COVID-19 pandemic on the general population of Saudi Arabia. Compr. Psychiatry.

[81] Loewenstein G.F., Weber E., Hsee C., Welch N. (2001). Risk as feelings. Psychol. Bull..

[82] Fessler D.M., Pillsworth E., Flamson T. (2004). Angry men and disgusted women: An evolutionary approach to the influence of emotions on risk taking. Organ. Behav. Hum. Decis. Processes.

[83] Slovic P., Finucane M., Peters E., MacGregor D. (2004). Risk as analysis and risk as feelings: Some thoughts about affect, reason, risk, and rationalit. Risk Anal. Int. J..

[84] Fan F., Ying Z., Yanyun Y., Mo L., Liu X. (2011). Symptoms of posttraumatic stress disorder, depression, and anxiety among adolescents following the 2008 Wenchuan earthquake in China. J. Trauma. Stress.

[85] Tausczik Y., Faasse K., Pennebaker J.W., Petrie K.J. (2012). Public anxiety and information seeking following the H1N1 outbreak: Blogs, newspaper articles, and Wikipedia visits. Health Commun..

[86] Yang C., Dillard J.P., Li R. (2018). Understanding fear of Zika: Personal, interpersonal, and media influences. Risk Anal..

[87] Finucane M.L., Alhakami A., Slovic P., Johnson S. (2000). The affect heuristic in judgments of risks and benefits. J. Behav. Decis. Mak..

[88] Wright W.F., Bower G.H. (1992). Mood effects on subjective probability assessment. Organ. Behav. Hum. Decis. Processes.

[89] Johnson E., Tversky A. (1983). Affect, generalisation, and the perception of risk. J. Personal. Soc. Psychol..

[90] Lerner J.S., Keltner D. (2000). Beyond valence: Toward a model of emotion-specific influences on judgement and choice. Cogn. Emot..

[91] Lerner J.S., Gonzalez R.M., Small D.A., Fischhoff B. (2003). Effects of fear and anger on perceived risks of terrorism: A national field experiment. Psychol. Sci..

[92] Costantino C., Restivo V., Ventura G., D’Angelo C., Randazzo M.A., Casuccio N., Palermo M., Casuccio A., Vitale F. (2018). Increased vaccination coverage among adolescents and young adults in the district of Palermo as a result of a public health strategy to counteract an ‘Epidemic Panic’. Int. J. Environ. Res. Public Health.

[93] Wakefield M.A., Loken B., Hornik R.C. (2010). Use of mass media campaigns to change health behaviour. Lancet.

[94] Kim J., Jung M. (2017). Associations between media use and health information-seeking behavior on vaccinations in South Korea. BMC Public Health.

[95] Alexander C.C. (2019). Media access is associated with knowledge of optimal water, sanitation and hygiene practices in Tanzania. Int. J. Environ. Res. Public Health.

[96] Bull S.S., Levine D.K., Black S.R., Schmiege S.J., Santelli J. (2012). Social media–delivered sexual health intervention: A cluster randomized controlled trial. Am. J. Prev. Med..

[97] Gough A., Hunter R.F., Ajao O., Jurek A., McKeown G., Hong J., Barrett E., Ferguson M., McElwee G., McCarthy M. (2017). Tweet for behavior change: Using social media for the dissemination of public health messages. JMIR Public Health Surveill..

[98] O’Leary S.T., Narwaney K.J., Wagner N.M., Kraus C.R., Omer S.B., Glanz J.M. (2019). Efficacy of a web-based intervention to increase uptake of maternal vaccines: An RCT. Am. J. Prev. Med..

[99] Lim J.S., Choe M.J., Zhang J., Noh G.Y. (2020). The role of wishful identification, emotional engagement, and parasocial relationships in repeated viewing of live-streaming games: A social cognitive theory perspective. Comput. Hum. Behav..

[100] Sarkar U., Le G.M., Lyles C.R., Ramo D., Linos E., Bibbins-Domingo K. (2018). Using social media to target cancer prevention in young adults. J. Med. Internet Res..

[101] Alhuwail D., Abdulsalam Y. (2019). Assessing electronic health literacy in the state of Kuwait: Survey of Internet users from an Arab state. J. Med. Internet Res..

[102] Stawarz K., Preist C., Coyle D. (2019). Use of smartphone apps, social media, and web-based resources to support mental health and well-being: Online survey. JMIR Mental Health.

[103] Privacy International (2020). Apps and COVID-19. https://privacyinternational.org/examples/apps-and-covid-19/.

[104] Amnesty International Bahrain, Kuwait and Norway Contact Tracing Apps among most Dangerous for Privacy. 16 June 2020. https://www.amnesty.org/en/latest/news/2020/06/bahrain-kuwait-norway-contact-tracing-apps-danger-for-privacy/.

[105] The New York Times (2020). Major Security Flaws Found in South Korea Quarantine App. https://www.nytimes.com/2020/07/21/technology/korea-coronavirus-app-security.html.

[106] O’Neill P.H. No, Coronavirus Apps Don’t Need 60% Adoption to Be Effectiv. https://www.technologyreview.com/2020/06/05/1002775/covid-apps-effective-at-less-than-60-percent-download/.

[107] Oldeweme A., Märtins J., Westmattelmann D., Schewe G. (2021). The role of transparency, trust, and social influence on uncUnertainty reduction in times of pandemics: Empirical study on the adoption of COVID-19 tracing apps. J. Med. Internet Res..

[108] Segal A. When China Rules the Web: Technology in Service of the State. Foreign Affairs.

[109] Galloway A., Bagshaw E. TikTok, WeChat to Face Australian Social Media Security Investigation. The Sydney Morning Herald.

[110] Lees J., Parker V.A. (2021). Hierarchy-Enhancing Misinformation: Social Dominance Motives Are Uniquely Associated with Republicans’ Belief in and Sharing of Election-Related Msinformation. psyarxiv.com/5jpgs.

[111] Aysan A.F., Ozer A., Seker M., Korkut C. (2020). Conceptualization of Uncertainty and Trust after the COVID-19 Outbreak1. Reflections on the Pandemic.

[112] Rousseau D.M., Sitkin S.B., Burt R.S., Camerer C. (1998). Not so different after all: A cross-discipline view of trust. Acad. Manag. Rev..

[113] Koufaris M., Hampton-Sosa W. (2004). The development of initial trust in an online company by new customers. Inf. Magement Syst..

[114] Moon M.J. (2020). Fighting COVID-19 with agility, transparency, and participation: Wicked policy problems and new governance challenges. Public Adm. Rev..

[115] Chanley V.A., Rudolph T., Rahn W.R. (2000). The origins of public trust in government. A time series analysis. Public Opin. Q..

[116] Keele L. (2007). Social capital and the dynamics of trust in government. Am. J. Political Sci..

[117] Stewart K.J. (2003). Trust transfer on the world wide web. Organ. Sci..

[118] Lu Y., Yang S., Chau P.Y., Cao Y. (2011). Dynamics between the trust transfer process and intention to use mobile payment services: A cross-environment perspective. Inf. Manag..

[119] Altmann S., Milsom L., Zillessen H., Blasone R., Gerdon F., Bach R., Kreuter F., Nosenzo D., Toussaert S., Abeler J. (2020). Acceptability of app-based contact tracing for COVID-19: Cross-country survey study. JMIR Mhealth Uhealth.

[120] Statista Number of Smartphone Users in Saudi Arabia from 2017 to 2025 (in Millions) 2020. https://www.statista.com/statistics/494616/smartphone-users-in-saudi-arabia/.

[121] Krejcie R.V., Morgan D.W. (1970). Determining sample size for research activities. Educ. Psychol. Meas..

[122] Alshira’h A.F., Alsqour M., Lutfi A., Alsyouf A., Alshirah M. (2020). A socio-economic model of sales tax compliance. Economies.

[123] Gefen D., Rigdon E.E., Straub D. (2011). Editor’s comments: An update and extension to SEM guidelines for administrative and social science research. MIS Q..

[124] Lutfi A. (2020). Investigating the moderating role of environmental uncertainty between institutional pressures and ERP adoption in Jordanian SMEs. J. Open Innov. Technol. Mark. Complex..

[125] Chin W.W., Vinzi V.E., Chin W., Henseler J., Wang H. (2010). How to write up and report PLS analyses. Handbook of Partial Least Squares.

[126] Lutfi A.A., Md Idris K., Mohamad R. (2017). AIS usage factors and impact among Jordanian SMEs: The moderating effect of environmental uncertainty. J. Adv. Res. Bus. Manag. Stud..

[127] Lutfi A., Alshira’h A.F., Alshirah M.H., Al-Okaily M., Alqudah H., Saad M., Ibrahim N., Abdelmaksoud O. (2022). Antecedents and Impacts of Enterprise Resource Planning System Adoption among Jordanian SMEs. Sustainability.

[128] Lutfi A., Alsyouf A., Almaiah M.A., Alrawad M., Abdo A.A., Al-Khasawneh A.L., Ibrahim N., Saad M. (2022). Factors Influencing the Adoption of Big Data Analytics in the Digital Transformation Era: Case Study of Jordanian SMEs. Sustainability.

[129] Alshirah M., Lutfi A., Alshira’h A., Saad M., Ibrahim N.M., Mohammed F. (2021). Influences of the environmental factors on the intention to adopt cloud based accounting information system among SMEs in Jordan. Accounting.

[130] Sarstedt M., Ringle C., Hair J. (2014). PLS-SEM: Looking back and moving forward. Long Range Plan..

[131] Lutfi A., Al-Okaily M., Alsyouf A., Alsaad A., Taamneh A. (2020). The impact of AIS usage on AIS effectiveness among Jordanian SMEs: A multi-group analysis of the role of firm size. Glob. Bus. Rev..

[132] Lutfi A. (2022). Understanding the Intention to Adopt Cloud-based Accounting Information System in Jordanian SMEs. Int. J. Digit. Account. Res..

[133] Lutfi A.A., Idris K.M., Mohamad R. (2016). The influence of technological, organizational and environmental factors on accounting information system usage among Jordanian small and medium-sized enterprises. Int. J. Econ. Financ. Issues.

[134] Hair J.F., Ringle C.M., Sarstedt M. (2011). PLS-SEM: Indeed a silver bullet. J. Mark. Theory Pract..

[135] Hair J.F., Sarstedt M., Hopkins L., Kuppelwieser V.G. (2014). Partial least squares structural equation modeling (PLS-SEM): An emerging tool in business research. Eur. Bus. Rev..

[136] Hair J.F., Ringle C.M., Sarstedt M. (2013). Partial least squares structural equation modeling: Rigorous applications, better results and higher acceptance. Long Range Plan..

[137] Preacher K., Hayes A. (2008). Asymptotic and resampling strategies for assessing and comparing indirect effects in multiple mediator models. Behav. Res. Methods.

[138] Sezgin E., Özkan-Yildirim S., Yildirim S. (2018). Understanding the perception towards using mHealth applications in practice: Physicians’ perspective. Inf. Dev..

[139] Deng Z., Hong Z., Ren C., Zhang W., Xiang F. (2018). What predicts patients’ adoption intention toward mHealth services in China: Empirical study. JMIR Mhealth Uhealth.

[140] Zhu Z., Liu Y., Che X., Chen X. (2018). Moderating factors influencing adoption of a mobile chronic disease management system in China. Inform. Health Soc. Care.

[141] Alam M.Z., Hoque M.R., Hu W., Barua Z. (2020). Factors influencing the adoption of mHealth services in a developing country: A patient-centric study. Int. J. Inf. Manag..

[142] Kissi J., Dai B., Dogbe C.S., Banahene J., Ernest O. (2020). Predictive factors of physicians’ satisfaction with telemedicine services acceptance. Health Inform. J..

[143] White D.B., Katz M.H., Luce J.M., Lo B. (2009). Who should receive life support during a public health emergency? Using ethical principles to improve allocation decisions. Ann. Intern. Med..

[144] Aslam U., Muqadas F., Imran M.K. (2018). Exploring the sources and role of knowledge sharing to overcome the challenges of organizational change implementation. Int. J. Organ. Anal..

[145] Muqadas F., Rehman M., Aslam U. (2017). Exploring the challenges, trends and issues for knowledge sharing: A study on employees in public sector universities. VINE J. Inf. Knowl. Manag. Syst..

[146] Naeem M. (2021). Do social media platforms develop consumer panic buying during the fear of COVID-19 pandemic. J. Retail. Consum. Serv..

[147] Alam T. (2020). mHealth Communication Framework using Blockchain and IoT Technologies. Int. J. Sci. Technol. Res..

[148] Fox G., Connolly R. (2018). Mobile health technology adoption across generations: Narrowing the digital divide. Inf. Syst. J..

[149] Jaber M.M., Alameri T., Ali M.H., Alsyouf A., Al-Bsheish M., Aldhmadi B.K., Ali S.Y., Abd S.K., Ali S.M., Albaker W. (2022). Remotely monitoring COVID-19 patient health condition using metaheuristics convolute networks from IoT-based wearable device health data. Sensors.

[150] AL-Mugheed K., Bayraktar N., Al-Bsheish M., AlSyouf A., Jarrar M., AlBaker W., Aldhmadi B.K. (2022). Patient Safety Attitudes among Doctors and Nurses: Associations with Workload, Adverse Events, Experience. Healthcare.

[151] Wheaton M.G., Abramowitz J.S., Berman N.C., Fabricant L.E., Olatunji B.O. (2012). Psychological predictors of anxiety in response to the H1N1 (swine flu) pandemic. Cogn. Ther. Res..

[152] Boudreaux E.D., Moon S., Baumann B.M., Carlos A., Camargo J., O’Hea E., Ziedonis D.M. (2010). Intentions to quit smoking: Causal attribution, perceived illness severity, and event-related fear during an acute health event. Ann. Behav. Med..

[153] Teo T.S., Srivastava S.C., Jiang L. (2008). Trust and electronic government success: An empirical study. J. Manag. Inf. Syst..

[154] Grayson K., Johnson D., Chen D. (2008). Is firm trust essential in a trusted environment? How trust in the business context influences customers. J. Mark. Res..

[155] Casaló L.V., Flavián C., Guinalíu M. (2007). The role of security, privacy, usability and reputation in the development of online banking. Online Inf. Rev..

[156] Al-Dmour H., Masa’deh R., Salman A., Abuhashesh M., Al-Dmour R. (2020). Influence of Social Media Platforms on Public Health Protection. J. Med. Internet Res..

